# Undiagnosed SARS-CoV-2 seropositivity during the first 6 months of the COVID-19 pandemic in the United States

**DOI:** 10.1126/scitranslmed.abh3826

**Published:** 2021-06-22

**Authors:** Heather Kalish, Carleen Klumpp-Thomas, Sally Hunsberger, Holly Ann Baus, Michael P. Fay, Nalyn Siripong, Jing Wang, Jennifer Hicks, Jennifer Mehalko, Jameson Travers, Matthew Drew, Kyle Pauly, Jacquelyn Spathies, Tran Ngo, Kenneth M. Adusei, Maria Karkanitsa, Jennifer A. Croker, Yan Li, Barry I. Graubard, Lindsay Czajkowski, Olivia Belliveau, Cheryl Chairez, Kelly R. Snead, Peter Frank, Anandakumar Shunmugavel, Alison Han, Luca T. Giurgea, Luz Angela Rosas, Rachel Bean, Rani Athota, Adriana Cervantes-Medina, Monica Gouzoulis, Brittany Heffelfinger, Shannon Valenti, Rocco Caldararo, Michelle M. Kolberg, Andrew Kelly, Reid Simon, Saifullah Shafiq, Vanessa Wall, Susan Reed, Eric W. Ford, Ravi Lokwani, John-Paul Denson, Simon Messing, Sam G. Michael, William Gillette, Robert P. Kimberly, Steven E. Reis, Matthew D. Hall, Dominic Esposito, Matthew J. Memoli, Kaitlyn Sadtler

**Affiliations:** 1 Trans-NIH Shared Resource on Biomedical Engineering and Physical Science, National Institute of Biomedical Imaging and Bioengineering, National Institutes of Health, Bethesda, MD 20894, USA.; 2 National Center for Advancing Translational Sciences, National Institutes of Health, Rockville, MD 20850, USA.; 3 Biostatistics Research Branch, National Institute of Allergy and Infectious Diseases, National Institutes of Health, Bethesda, MD 20894, USA.; 4 Clinical Studies Unit, Laboratory of Infectious Diseases, National Institute of Allergy and Infectious Diseases, National Institutes of Health, Bethesda, MD 20894, USA.; 5 Clinical and Translational Science Institute, University of Pittsburgh, Pittsburgh, PA 15213, USA.; 6 Clinical Monitoring Research Program Directorate, Frederick National Laboratory for Cancer Research, Frederick, MD 21702, USA.; 7 Protein Expression Laboratory, NCI RAS Initiative, Frederick National Laboratory for Cancer Research, Frederick, MD 21702, USA.; 8 Section on Immuno-Engineering, National Institute of Biomedical Imaging and Bioengineering, National Institutes of Health, Bethesda, MD 20894, USA.; 9 Center for Clinical and Translational Science, School of Medicine, University of Alabama at Birmingham, Birmingham, AL 35294, USA.; 10 Joint Program in Survey Methodology, Department of Epidemiology and Biostatistics, University of Maryland College Park, College Park, MD 20742, USA.; 11 Division of Cancer Epidemiology and Genetics, Biostatistics Branch, National Cancer Institute, National Institutes of Health, Bethesda, MD 20894, USA.; 12 Laboratory of Immunoregulation, National Institute of Allergy and Infectious Diseases, National Institutes of Health, Bethesda, MD 20894, USA.; 13 Clinical Research Directorate, Frederick National Laboratory for Cancer Research, Leidos Biomedical Research Inc., Frederick, MD 21702, USA.; 14 Division of Clinical Research, National Institute of Allergy and Infectious Diseases, National Institutes of Health, Bethesda, MD 20894, USA.

## Abstract

Asymptomatic SARS-CoV-2 infection and delayed implementation of diagnostics have led to poorly defined viral prevalence rates in the United States and elsewhere. To address this, we analyzed seropositivity in 9089 adults in the United States who had not been diagnosed previously with COVID-19. Individuals with characteristics that reflected the U.S. population (*n* = 27,716) were selected by quota sampling from 462,949 volunteers. Enrolled participants (*n* = 11,382) provided medical, geographic, demographic, and socioeconomic information and dried blood samples. Survey questions coincident with the Behavioral Risk Factor Surveillance System survey, a large probability-based national survey, were used to adjust for selection bias. Most blood samples (88.7%) were collected between 10 May and 31 July 2020 and were processed using ELISA to measure seropositivity (IgG and IgM antibodies against SARS-CoV-2 spike protein and the spike protein receptor binding domain). The overall weighted undiagnosed seropositivity estimate was 4.6% (95% CI, 2.6 to 6.5%), with race, age, sex, ethnicity, and urban/rural subgroup estimates ranging from 1.1% to 14.2%. The highest seropositivity estimates were in African American participants; younger, female, and Hispanic participants; and residents of urban centers. These data indicate that there were 4.8 undiagnosed SARS-CoV-2 infections for every diagnosed case of COVID-19, and an estimated 16.8 million infections were undiagnosed by mid-July 2020 in the United States.

## Elucidating seroprevalence in COVID-19

Symptoms of SARS-CoV-2 infection range from completely asymptomatic, to those of a common cold, to a drop in oxygen saturation and lung function, and death in some patients. To evaluate the proportion of the U.S. population who had an undiagnosed infection during the first wave of the COVID-19 pandemic, we measured antibody prevalence in study participants who had not previously been diagnosed with a SARS-CoV-2 infection. By mid-July of 2020, 16.8 million people had an undiagnosed SARS-CoV-2 infection, almost five times the rate of diagnosed infections.

## Teaser

16.8 million SARS-CoV-2 infections in the US went undiagnosed in the first 6 months of the pandemic compared to 3.5 million diagnosed infections.

## INTRODUCTION

Coronavirus disease 2019 (COVID-19), the disease caused by severe acute respiratory syndrome coronavirus type 2 (SARS-CoV-2) infection, presents with a spectrum of illness ranging from asymptomatic to severe disease. As with most respiratory viral diseases, it is difficult to estimate the true prevalence of the disease during a pandemic and the extent of its spread is only known after extensive study (*1*–*3*). Most patients infected with SARS-CoV-2 develop robust antibody responses against the viral spike protein, nucleocapsid protein, and the envelope protein that can be detected by serological testing (*4*–*8*). Antibodies against spike protein persist for months and can neutralize SARS-CoV-2 (*9*). Frequently, these neutralizing antibodies bind to the receptor binding domain (RBD) of the spike protein, but antibodies against the spike protein S2 domain have also been observed (*10*–*15*).

To characterize the spread of SARS-CoV-2 infection in the United States, we evaluated seropositivity in a national survey of participants who had not previously been diagnosed with SARS-CoV-2 infection. We used quota sampling from a large pool of volunteers (*n* = 462,949) to obtain a representative sample (*n* = 9089) and performed statistical weighting to generate prevalence estimates that revealed the extent of SARS-CoV-2 infection in the general population. To ensure accurate classification of seropositivity, we used our dual-antigen enzyme-linked immunosorbent assay (ELISA) protocol that evaluated immunoglobulin G (IgG) and IgM antibodies against both the full viral spike protein ectodomain and the RBD (*8*, *16*).

## RESULTS

### Enrollment and demographic representation

**Fig. 1:**
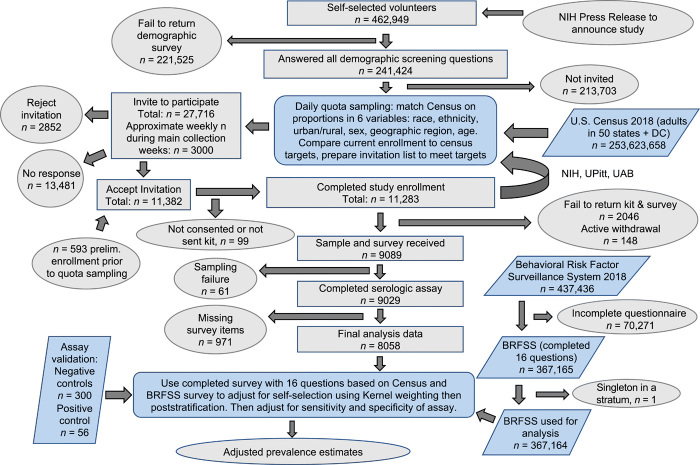
SARS-CoV-2 serosurvey study overview and statistical workflow. A flow chart of participant recruitment through data analysis displays steps in data acquisition and lists participant attrition. Ovals show the start and end of data analysis or data acquisition; gray rectangles indicate subsets of participants in this study; blue parallelograms represent individuals from outside data sets that contributed to adjusted prevalence estimates; blue rounded rectangles present analysis processes.

Recruitment took place from 1 April 2020 to 4 August 2020. During that time, 11,283 participants were enrolled from a pool of 241,424 volunteers in the United States (50 states and the District of Columbia). Of these participants, 214 had blood collected via venipuncture and 11,069 were sent volumetric dried blood microsamplers (absorbent polymer, 20-μl collection volume). More than 80% of the microsamplers were returned (9089 participants). Ultimately, 9028 participant blood samples were analyzed using ELISA for the presence of anti–SARS-CoV-2 spike protein antibodies. Of those, 8058 participants had a complete clinical questionnaire and were included in the weighted analysis (Fig. 1). Most blood sample collection (>88%) occurred within the 11-week period between 10 May and 31 July 2020 (figs. S1 and S2). The six major demographic factors used in participant selection are summarized in Table 1. Participant sampling was representative of the U.S. population. When expanded to include the additional 10 demographic or health-related factors captured by the Behavioral Risk Factor Surveillance System (BRFSS), many factors were well matched, but there were some differences, for example, our sample population was more highly educated, had higher employment rates, and had better access to health care compared to the general U.S. population (Table 1).

**Table 1:** Characteristics of the serosurvey population compared to the U.S. population. Census and BRFSS (2018) data on selection criteria were used for quota-based sampling in our SARS-CoV-2 serosurvey. Other values from BRFSS were used for statistical weighting. The table shows comparisons between the estimated proportion of the U.S. population in each category according to weighted BRFSS data compared to our sample population in the SARS-CoV-2 serosurvey. NLF, not in the labor force (student, retired, unable to work, refused to answer, not asked/missing).

	**U.S. population (BRFSS, census survey)**			**SARS-CoV-2 serosurvey population**	
	***n***	**%**	**Weighted** **(%)**	***n***	**%**
Selection criteria					
Region					
North East	91,307	21.19	17.6	1,508	16.7
Midwest	67,110	15.57	16.97	1,445	16.01
Mid-Atlantic	80,979	18.79	16.91	1,833	20.3
South/Central	60,482	14.03	15.35	1,293	14.32
Mountain/Southwest	86,204	20	15.89	1,392	15.42
West/Pacific	44,866	10.41	17.27	1,557	17.25
Age group					
18–45	125,081	28.59	46	3,837	42.51
45–70	207,749	47.49	39.84	3,783	41.91
70–95	104,605	23.91	14.17	1,407	15.59
Sex					
Male	197,411	45.24	48.66	4,318	47.83
Female	238,911	54.76	51.34	4,710	52.17
Urban/rural					
Urban	365,714	84.9	93.48	8,550	94.78
Rural	65,234	15.1	6.52	471	5.22
Race					
White only	345,710	81	73.41	6,986	77.4
Black only	37,862	8.87	12.9	830	9.2
Others	43,219	10.13	13.69	1,210	13.41
Ethnicity					
Hispanic	36,941	8.53	17.06	1,495	16.56
Not Hispanic	395,931	91.47	82.94	7,532	83.44
					
Additional weighting criteria					
Children					
Yes	113,408	26.21	35.81	2,943	32.88
No	319,281	73.79	64.19	6,009	67.12
Education					
<=HS	151,606	34.79	41.07	240	2.68
College	119,979	27.53	30.88	1,284	14.35
>=College	164,229	37.68	28.05	7,422	82.96
Homeowner					
Own	305,545	70.36	66.49	6,635	74.12
Rent	107,208	24.69	27.32	1,861	20.79
Other	21,535	4.96	6.19	456	5.09
Employment					
Employed	219,493	50.75	57.74	6,364	71.09
NLF	174,920	40.45	31.38	2,129	23.78
Unemployed	38,053	8.8	10.88	459	5.13
Health insurance					
Yes	400,028	91.86	87.85	8,697	97.31
No	35,433	8.14	12.15	240	2.69
Flu vaccinated					
Yes	234,727	59	50.62	6,198	73.73
No	163,124	41	49.38	2,208	26.27
Cardiovascular disease					
Yes	52,284	12.07	9.07	354	3.98
No	380,985	87.93	90.93	8,541	96.02
Pulmonary disease					
Yes	84,102	19.33	18.53	1,671	18.96
No	350,913	80.67	81.47	7,140	81.04
Immune disease					
Yes	170,115	39.14	29.29	2,039	23.1
No	264,571	60.86	70.71	6,787	76.9
Diabetes					
Yes	60,703	13.9	11.41	482	5.41
No	375,876	86.09	88.59	8,430	94.59
					

### Estimates of seroprevalence

**Fig. 2:**
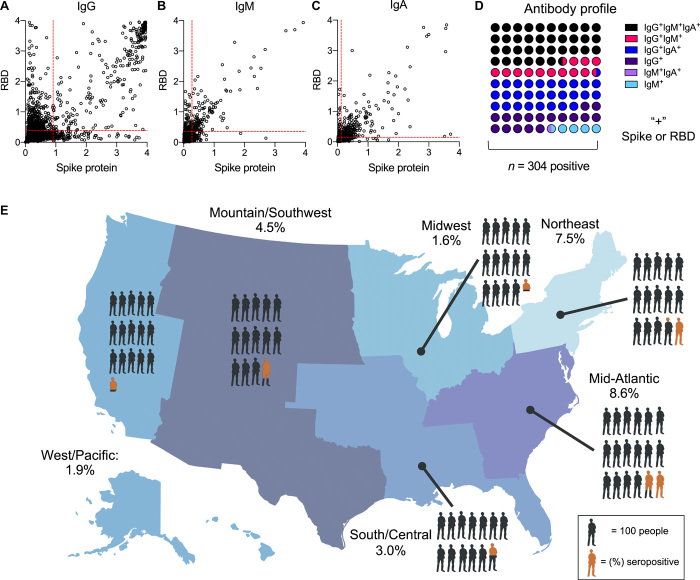
Geographic distribution of undiagnosed seropositivity in the United States from May to July 2020. Raw serology data for (**A**) IgG, (**B**) IgM, and (**C**) IgA against SARS-CoV-2 spike protein and the receptor binding domain (RBD) of spike protein are displayed. Cut points for positivity are shown as red dashed lines; data are optical density (OD). (**D**) Serologic phenotype of antibody presence in 304 seropositive participants. (**E**) The map of the United States displays seropositivity in the six regions surveyed: Northeast: ME, NH, VT, MA, NY, CT, RI, PA, and NJ, 7.5% (95% CI, 3.9% to 12.4%); Midwest: MN, IA, WI, IL, IN, MI, and OH, 1.6% (95% CI, 0.3% to 2.4%); Mid-Atlantic: MD, DE, DC, VA, WV, KY, TN, NC, SC, and GA, 8.6% (95% CI, 2.6% to 18.9%); South/Central: FL, MS, AL, LA, AR, MO, KS, and OK, 3.0% (95% CI, 1.2% to 5.0%); Mountain/Southwest: TX, NM, AZ, CO, UT, WY, NE, SD, ND, MT, and ID, 4.5% (95% CI, 1.3% to 9.5%); West/Pacific: WA, OR, NV, CA, AK, and HI, 1.9% (95% CI, 0.2% to 3.8%). Each person in (E) represents 100 participants; orange represents weighted prevalence estimate within the geographic region.

There were 304 seropositive participants in the analysis set (Fig. 2). This gave a weighted estimate of 4.6% of the undiagnosed adults in the U.S. population who were seropositive for SARS-CoV-2 infection [95% confidence interval (CI), 2.6% to 6.5%, *n* = 8058 complete testing and survey]. Using this average rate over the study period, we estimated that there were 4.8 undiagnosed SARS-CoV-2 infections for each diagnosed case over the course of the study (95% CI, 2.8 to 6.8). Among seropositive participants, 36.51% were IgG^+^IgM^+^IgA^+^, 28.29% were IgG^+^IgM^−^IgA^+^, 17.11% were IgG^+^IgM^−^IgA^−^, 13.16% were IgG^+^IgM^+^IgA^−^, 4.28% were IgG^−^IgM^+^IgA^−^, and 0.66% were IgG^−^IgM^+^IgA^+^ (Fig. 2, A to D, and fig. S3). There were variations in antibody profiles across different demographic groups, specifically anti-spike protein and anti-RBD IgG antibodies (figs. S4 and S5).

**Fig. 3:**
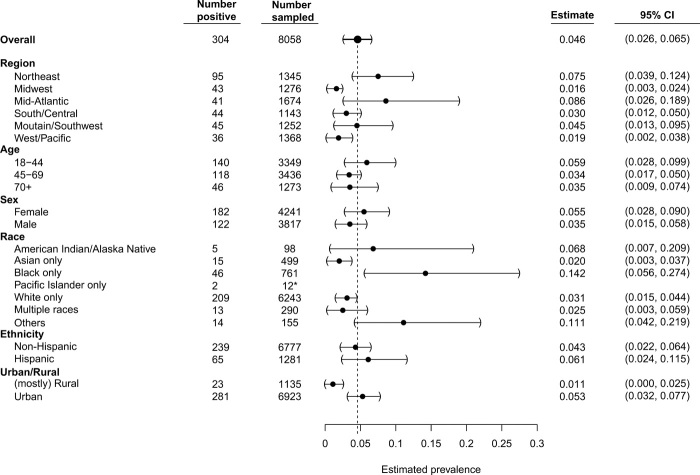
Undiagnosed SARS-CoV-2 seroprevalence in the main demographic categories. Six main categories were used during quota-based sampling: region, age, sex, race, ethnicity, and urban/rural. Seropositivity estimates of blood samples that had a full clinical questionnaire completed and successful sampling are shown. Data are weighted estimates ± 95% CIs. Black dashed vertical line, weighted national seroprevalence estimate; *, *n* value too low to make a proper weighted estimate so raw positivity is displayed.

We found regional variations in seroprevalence estimates across the United States (Figs. 2E and 3). The Northeast and Mid-Atlantic regions showed the highest rates of seropositivity, whereas the lowest seropositivity was in the Midwest. Urban areas were estimated to have higher point estimates of seropositivity (5.3%) compared to rural areas (1.1%) at the time blood samples were collected. Estimates of seroprevalence were calculated for other demographic subgroups (Fig. 3). The youngest age group, 18 to 44 years, had the highest estimated seropositivity (5.9%). Estimated seroprevalence for females was 5.5% and was 3.5% for males. The seroprevalence estimate for African Americans was highest at 14.2% followed by participants who self-identified as other/unlisted race (11.1%), American Indian/Alaska Native (6.8%), followed by White/Caucasian (3.1%), whereas those identifying as Asian displayed the lowest seroprevalence estimate (2.0%).

**Fig. 4:**
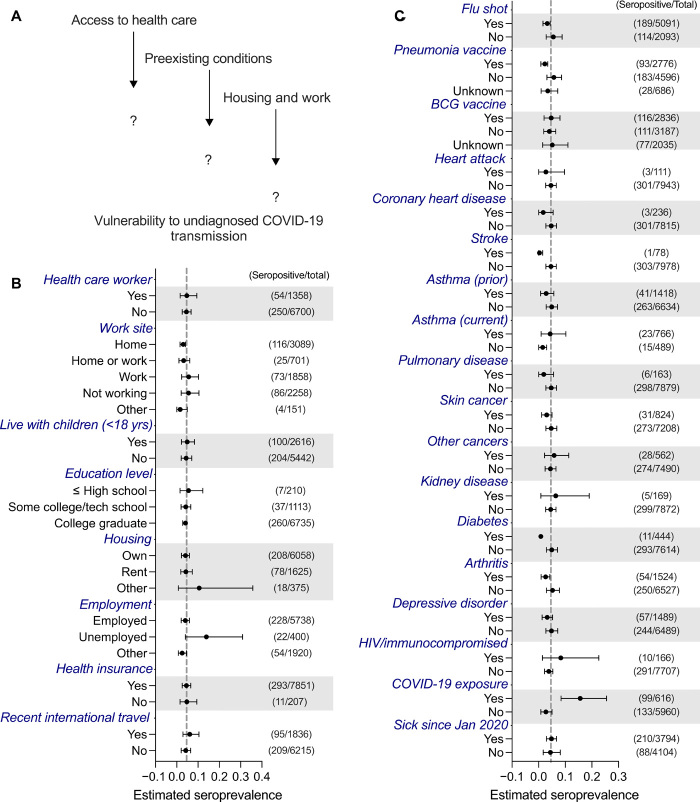
Seroprevalence estimates according to socioeconomic and health characteristics. (**A** to **C**) Evaluation of the effect of nondemographic traits on seroprevalence estimates for blood samples that had a full clinical questionnaire completed and successful sampling. Nondemographic traits included (B) socioeconomic and (C) health characteristics. Data are weighted estimates ± 95% CIs. Gray dashed vertical line, weighted national seroprevalence estimate.

Participants who reported a known exposure to a SARS-CoV-2–infected individual had a higher seroprevalence estimate (15.6%) compared to those who did not (2.7%). In comparison to the national average (4.6%), those who worked from home had a lower seropositivity estimate of 3.0%. Those who reported previous vaccination (for influenza 3.2% or pneumonia 2.3%) had a lower likelihood of undiagnosed seropositivity. Those who had health conditions associated with poor outcomes in SARS-CoV-2 infection, including coronary heart disease, asthma, and diabetes, displayed lower rates of seropositivity (Fig. 4). Other health conditions were also correlated with a decreased seropositivity rate such as skin cancer, stroke, or arthritis.

Our results estimate that as of July 2020, there were about 4.79 undiagnosed infections (95% CI, 2.76 to 6.82; fig. S6) for every identified case of COVID-19, suggesting a potential 16.8 million undiagnosed infections by July 2020 in addition to the reported 3.5 million diagnosed cases in the United States. These data suggest that a higher level of infection-induced immunity exists in the U.S. population than previously predicted.

## DISCUSSION

These results, including the subgroup analysis, provide us a previously undescribed view into the spread of the COVID-19 pandemic by more clearly identifying the large numbers of individuals with undiagnosed infections during the initial months of the pandemic. These data are of great importance as we consider the impact vaccination may have on the future course of the pandemic and plan for current and future available vaccines to be administered. In addition, these data can also help us to better assess the public health measures taken during the pandemic and how to take the best approaches forward during any future public health emergencies.

This study demonstrates that spread of the SARS-CoV-2 virus in the United States during the first 6 months of the pandemic was more widespread than has been suggested by data reporting diagnostic test-confirmed cases. Similar to responses to other respiratory viruses, such as influenza, many individuals develop asymptomatic or mild disease that is not medically attended and therefore never diagnosed. Our findings indicate that there are nearly five individuals with a previous asymptomatic infection for every diagnosed case of COVID-19. Furthermore, patterns of our seroprevalence data match well with those of diagnosed cases reported during a similar time frame (*17*). For example, the greater seropositivity estimated in densely populated urban areas follows the observed initial spread of SARS-CoV-2. In comparison to the national average, we found that the Midwest, South, and West had lower seroprevalence rates during the study time frame, which preceded a substantial increase in SARS-CoV-2 infections in these regions detected by viral testing.

Our data suggest that the youngest age group had the highest undiagnosed seroprevalence, which is consistent with observations that they display less severe symptoms than older patients (*18*). We also found higher undiagnosed seroprevalence in females, possibly suggesting a higher risk for asymptomatic disease. Participants with chronic diseases that are more likely to be associated with severe clinical manifestations of COVID-19, including diabetes, heart disease, and asthma, had a lower prevalence of asymptomatic SARS-CoV-2 infection in comparison to the national average. Those with known exposure to SARS-CoV-2–infected individuals had a higher estimated incidence of undiagnosed seropositivity. We also found that African American and Hispanic participants had higher undiagnosed seropositivity, correlating with national data on disease burden in these subgroups.

Our study reports a representative population sample across the United States and evaluated regional, demographic, and socioeconomic differences in the prevalence of asymptomatic SARS-CoV-2 infection. In contrast, other reports of seroprevalence data focus on specific groups of individuals or geographic locations, such as dialysis patients or individuals who reported for blood draws that may be biased toward those needing medical care during the pandemic (*19*–*36*). These previous studies came within the range of our estimate of undiagnosed cases when considering the additional diagnosed cases within the same time frame. Our results provide new insight into the spread of SARS-CoV-2, estimating the national undiagnosed exposure rate to illuminate the scope of infection during the first 6 months of the pandemic. As expected, given delayed arrival in different geographic areas such as the Midwest and rural South, undiagnosed infection estimates varied by region, with the Mid-Atlantic region having the largest proportion of undiagnosed infections in comparison to diagnosed cases. Given the high point estimate of undiagnosed seropositivity in younger participants, lower point estimates in individuals with preexisting conditions such as diabetes, and the vaccine rollout starting with older persons and those at risk, we could see a faster onset of herd immunity due to these undiagnosed infections in populations that are in lower priority groups for vaccination. Young and healthy individuals, such as those under the age of 16 who were not eligible for the first wave of vaccines in the United States and those under 12 who are still ineligible, could serve as an asymptomatic reservoir for viral mutations leading to increased transmissibility or vaccine escape mutations, which has been shown in unvaccinated children and adults with viral persistence (*37*). Further long-term studies of immunity in the population will be necessary to understand durability of the immune response to the vaccine versus infection, how infection-induced immunity affects vaccine response and performance, and whether herd immunity can play a role in controlling the spread of SARS-CoV-2. In addition, further subgroup analysis of these data will be useful in clarifying the spread of disease in the presence of public health measures and how we may be able to refine and further target those measures in the future.

Our study has several limitations. First, although extensive statistical adjustments were made, our study cohort is based on a nonrandom volunteer sample, which can have selection bias. Traditional random sampling studies using probability sampling design may have low response rates, calling into question the advantages of that practice (*38*, *39*). Our study population also exhibited some differences from the general U.S. population, such as higher education level and access to health care that had to be adjusted for with statistical weighting. Larger sample sizes would allow us to make more detailed estimates, although potentially at the cost of how representative the population is. We used both census and behavioral data to weight our results, although it is possible that there are variables associated with disease transmission that were not accounted for in our weighting. Although we used extensive validation methods on our ELISA (*8*) for seropositivity designations, we used historical serum samples and convalescent post-infection samples because dried blood was unavailable from historical samples on the collection devices. Future cross-verification with an independent analyte, such as the nucleocapsid protein, could prove useful, although antibodies to nucleocapsid fade and would require correction for antibody decay.

Our data suggest a larger spread of the COVID-19 pandemic in the United States during the first 6 months than originally thought. Our findings have implications for understanding SARS-CoV-2 spread, epidemiological characteristics of spread, and prevalence in different communities and could have a potential impact on decisions involved in vaccine rollout. Continued large-scale surveillance of SARS-CoV-2 immunity is in progress, discriminating infection-based and vaccine-induced antibody responses. Mathematical models are being generated to understand the pandemic, vaccine performance, and public health measure efficacy and to provide insight into the best approach for handling the next virus with pandemic potential.

## MATERIALS AND METHODS

### Study design

This study was designed to determine the seroprevalence of anti–SARS-CoV-2 antibodies in adults 18 years of age or older in the United States who had not been previously diagnosed with COVID-19. The primary endpoint was the weighted estimate of seroprevalence in the United States. Secondary endpoints were weighted estimates for subgroups categorized by demographics or risk factors. An initial period enrolled a convenience sample of 593 volunteers before the quota sample. Participants across the United States (50 states and District of Columbia) were then enrolled through telephone consent from a pool of volunteers who provided basic demographic data in response to the study announcement. Recruitment calls were made from three sites: National Institute of Allergy and Infectious Diseases (NIAID) Laboratory of Infectious Diseases Clinical Studies Unit, the University of Pittsburgh Clinical and Translational Science Institute (CTSI), and the University of Alabama at Birmingham Center for Clinical and Translational Science (CCTS). The selection of participants is described below. Selected participants were contacted by the study team, consented, and sent a blood microsampling kit and questionnaire in the online REDCap platform (project-redcap.org). For a small subset of participants (*n* = 214) working on the National Institutes of Health (NIH) campus, serum was collected by venipuncture.

This serosurvey clinical study (ClinicalTrials.gov NCT04334954) is ongoing and will follow the same cohort of participants over time to evaluate seroprevalence and antibody profiles in comparison to the demographic, health, and socioeconomic data provided by each participant. This study was approved by the NIH Institutional Review Board and conducted in accordance with the provisions of the Declaration of Helsinki and Good Clinical Practice guidelines. All participants provided verbal informed consent before enrollment.

### Participant selection

The study was advertised online through an official NIH Press Release that linked to an email address to volunteer for selection in the study (www.niaid.nih.gov/news-events/nih-begins-study-quantify-undetected-cases-coronavirus-infection). This press release was subsequently publicized by local and national news outlets and covered via broadcast television news, print news, and internet news articles. All volunteers were emailed an initial survey to collect basic demographic characteristics. Survey responses were de-identified and aggregated by subcategory of state, type of locality approximated from zip codes, age, sex, race, and ethnicity (Fig. 1). Target sample sizes for these subcategories were determined from the U.S. census and were updated every evening based on the characteristics of people who had already enrolled to assure that individuals in each subcategory were enrolled evenly over time. Within each subcategory, participants were initially assigned a selection probability calculated from the target number as a proportion of the available pool. Specific subcategories that had insufficient numbers were aggregated to estimate their impact on the overall distribution of the six main characteristics. If a particular characteristic had insufficient numbers, sample probabilities were boosted for volunteers who had the characteristic. For each day’s call list, the most representative of 20,000 randomly generated lists was used, each list drawn without replacement from the volunteer pool based on the sampling probabilities previously defined. Representativeness was assessed by estimating a weighted sum of squared differences from the desired targets and picking the list with the lowest deviation. Unselected participants were eligible to be called at a later date. This algorithm is designed such that each cohort of invited participants is representative of the diversity of the U.S. population with respect to the six sampling variables (see section S4).

### Blood sample collection

Participants provided blood samples by mail using a Mitra microsampling kit (Neoteryx, Torrance, CA) or standard venipuncture. Microsampling kits contained visual instructions on the sampling process, bandages, gauze, lancets, and four 20-μl microsampling devices for a total collection of 80 μl of whole blood. Participants used the lancet to draw blood from their fingertip and collect blood onto each of the four microsamplers. Participants returned the dried microsamplers with desiccant via overnight shipping. Those who underwent venipuncture did so in the NIH Clinical Center phlebotomy laboratory, where 18 ml of blood was collected in a serum separator and whole blood tube. Once received in the laboratory, serum samples were processed, and microsamplers were stored dry at −80°C until elution and analysis.

### Serologic assays

Antibodies from samples were analyzed using ELISA as previously described (*8*, *40*–*42*). To maintain longitudinal quality control and ensure that the assays remained stable across multiple months of assay implementation, positive and negative controls were included on each assay plate and monitored for stability (fig. S7). Seropositivity cut points were defined by evaluating 300 true-negative samples and 56 true-positive samples. Positivity thresholds were based on the mean optical density (absorbance) plus 3 SDs (see the Supplementary Materials for details). The final criterion of a Spike^+^ and RBD^+^ for any combination of IgG or IgM gave estimated sensitivity and specificity of 1, with raw values for recombinant antibody results reported in fig. S8 and table S1. In addition, IgA was evaluated via previously described ELISA to further phenotype the participant’s serologic status. Raw sample positivity data by state can be found in fig. S9.

### Statistical analysis

The iterative quota sampling (described in the “Participant selection” section) that we used continuously matched the proportion of people in the study with the census estimated proportion of people in the United States on six variables (Table 1 and Fig. 1). This ensured that each periodic sample of participants over the course of the study was representative, and the time effects of the pandemic were approximately independent of those six variables (fig. S2). Each participant was asked demographic and health-related questions that matched those on the BRFSS survey, a large probability-based national survey (*43*). Responses to those matching questions were used with BRFSS survey data to adjust estimators to account for important criteria that may be related to both selection probability and seropositivity but were not accounted for in our quota sampling. Those adjusted estimators used weighting based on the propensity of being a quota sample versus a BRFSS sample participant and poststratification to U.S. census data. Weighting additionally accounted for sensitivity and specificity. CIs were calculated for the final seroprevalence estimates accounting for both the variability of the weighting and of the sensitivity and specificity adjustment. The ratio of undiagnosed SARS-CoV-2 infections to diagnosed cases of COVID-19 was estimated as the final seroprevalence estimate times a factor calculated from the daily national population and diagnosed cases. Detailed statistical methods are provided in the Supplementary Materials. The main computer code used in this study is available at: https://zenodo.org/record/4958017#.YMkzYpNKh26. Sources used for analysis can be found in (*8*, *38*, *39*, *43*–*56*).

## SUPPLEMENTARY MATERIALS

stm.sciencemag.org/cgi/content/full/13/601/eabh3826/DC1
Statistical Methods Figs. S1 to S9 Table S1

## Appendix

View/request a protocol for this paper from *Bio-protocol*.
